# L-lysine improves pork quality during postmortem aging: insights into meat quality, protein properties, and enzyme activities

**DOI:** 10.5713/ab.24.0901

**Published:** 2025-04-28

**Authors:** Xiuyun Guo, Shuangyi Xu, Chao Fu, Xiangren Meng

**Affiliations:** 1 School of Tourism and Cuisine, Yangzhou University, Yangzhou, China; 2 Key Laboratory of Chinese Cuisine Intangible Cultural Heritage Technology Inheritance, Ministry of Culture and Tourism, Yangzhou, China

**Keywords:** Enzyme Activities, L-lysine, Meat Quality, Postmortem Aging, Proteins Properties

## Abstract

**Objective:**

This study aimed to investigate the intrinsic relationships between meat quality, protein properties, and enzyme activities of pork *longissimus dorsi* treated with L-lysine (Lys) during postmortem aging.

**Methods:**

The pork samples were collected from 18 twelve-month-old crossbred pigs (120 kg, Duroc×Long White Large×White, *longissimus dorsi* muscle) in three batches of six samples each. The meat was then immediately placed in a thermal container and transported to the laboratory within 1 hour for subsequent processing at 4°C. After removing fat and connective tissue, the pork was cut into 20 g±1 g meat blocks. Then, the samples were vacuum sealed and left in a freezer (4°C) for 0, 1 and 3 days.

**Results:**

The results showed that Lys addition (0.10%, 0.15%, and 0.20%) improved pork quality (water-holding capacity and tenderness). On the third day of aging, the shear force values reached their lowest levels (p<0.05), measuring 38.21 N, 34.04 N, and 30.94 N for the respective treatment groups. In addition, the postmortem addition of Lys significantly increased the myofibrillar fragmentation index and actomyosin solubility during pork aging (p<0.05), with maximum values of 105.07% and 90.35%, respectively. Meanwhile, microscopic structure and electrophoresis results indicated that Lys treatment disrupted the muscle fiber structure, promoted the degradation of myofibrillar proteins (MPs) and dissociation of actomyosin. Furthermore, with increasing Lys addition, calpain-1 activity, caspase-3 activity, and Ca^2+^-ATPase activity in the muscle significantly increased (p<0.05), reaching maximum values of 17.46 ng/mL, 55.68 μg/mL, and 2.79 μmol (Pi)/min·mg protein, respectively. The activation of these enzymes promoted the dissociation and hydrolysis of key structural proteins.

**Conclusion:**

Lys improved pork quality by increasing calpain-1, caspase-3, and Ca^2+^-ATPase activity during the postmortem aging, thereby promoting the degradation of MPs and the dissociation of the actomyosin.

## INTRODUCTION

Global pork consumption levels have shown an increasing trend, with China accounting for approximately half of global pork production and consumption [1]. It is well known that pork undergoes a series of biochemical and physiological reactions after slaughter, including apoptosis, glycolysis and protein degradation, which primarily determine pork quality and influence consumer purchasing preferences [2,3]. Consequently, it has attracted considerable attention from researchers worldwide aiming to enhance pork quality by targeting the physiological and biochemical reactions during postmortem aging.

As known, the degradation of key structural proteins (e.g., troponin-T, desmin, actin) and the disruption of myofibrils, induced by endogenous protease such as calpains and caspases, primarily contribute to the development of meat tenderness and water-holding capacity (WHC) during postmortem aging [4–6]. In addition, the formation of the actomyosin (AM) complex, driven by Ca^2+^-mediated adenosine triphosphate (ATP) consumption, leads to muscle contraction and increased hardness [7]. Hence, the dissociation of AM into actin and myosin may also contribute to the enhancement of meat quality. In summary, promoting the degradation of myofibrillar proteins (MPs) and the dissociation of AM during postmortem aging is effective for enhancing meat quality.

Over the past decades, the application of L-lysine (Lys) in meat and meat products has causing great attention due to its good performance in modifying sensory defects of low sodium or non-chloride salt-substituted meat products [8,9]. The studies find that Lys alters the secondary structures and tertiary structures of MPs and myosin, contributing to the degradation of MPs/myosin filaments, thereby improving the functional properties of meat proteins (e.g., gelation and emulsification) and the quality of meat and meat product (tenderness, color, and WHC) [10].

In addition, some reports indicate that Lys promotes the dissociation of AM and alters the thermal behavior and microstructure of AM gels by increasing surface hydrophobicity and active sulfhydryl groups, ultimately improving gel characteristics [5,11]. Furthermore, it was found that Lys can increase the sarcomere length and the myofibrillar fragmentation index (MFI) of chicken breast, which may be resulted from the accelerated degradation of troponin-T [11]. In short, Lys has the potential to improve meat quality via regulating the degradation of MPs and the dissociation of AM during postmortem aging. Nevertheless, to date, there is few studies focused on it.

The present study investigates the effects of different concentrations of Lys on pork quality during postmortem aging, the MPs degradation, Ca^2+^-ATPase activity, calpain-1 activity and caspase-3 activity are determined to elucidate the mechanisms by which Lys enhances pork quality during postmortem aging.

## MATERIALS AND METHODS

### Materials

The pork was obtained from 18 twelve-month-old crossbred pigs (Duroc×Long White Large×White, *longissimus dorsi* muscle) in three batches of six samples each, sourced from Yangzhou Dingxin Food Group (Yangzhou, China). The meat was then immediately placed in a thermal container and transported to the laboratory within 1 hour for subsequent processing at 4°C. Lys (Cas: 56-87-1) and all other chemicals used were of analytical grade and procured from Sigma-Aldrich.

### Sample processing

After removing fat and connective tissue, the pork was cut into 20 g±1 g meat blocks. Lys solutions of varying concentrations (0.10%, 0.15%, 0.20%) were prepared using deionized water (4°C, pH 7.0). The samples were marinated in deionized water (Control) and different Lys solutions (W/V: 1:4, 4°C) for 60 min. Then, the samples were vacuum sealed and left in a freezer (4°C, YC-800; Zhongmeida Refrigeration Technology) for 0, 1 and 3 days. The above operations are in the 4°C constant temperature room.

### pH

The pH of pork samples was measured at 0, 1, and 3 days of aging using a temperature-compensated portable pH meter (Testo 205; Testo). Probes were randomly inserted into six locations, and the pH was recorded after each measurement.

### Water-holding capacity

*Cooking loss:* The cooking loss (CL) was determined by the method of Xu et al [2]. The CL was calculated using the following formula:


(1) $$\text{Cooking loss}/\%=\frac{M_{1}-M_{2}}{M_{1}}\times 100$$

Where M_1_: pre-cooking quality, M_2_: post-cooking quality.

*Pressurization loss:* The samples were sliced into 1 cm thick pieces along the direction of the muscle fibers. Each sample was then placed between two pads, each consisting of 18 layers of medium-speed qualitative filter paper. The samples were positioned on an unconfined compression platform (SM-8007; L-TEST Electronic Technology), and a 35 kg weight was gradually applied for 3 min. The sample weights before and after compression were recorded as m_1_ and m_2_, respectively. The pressurization loss (PL) was calculated using the following formula:


(2) $$\text{Pressurization loss}/\%=\frac{m_{1}-m_{2}}{m_{1}}\times 100$$

### Shear force

The shear force was measured using the shear force apparatus (C-LM_3B_; Yangzhou University). A circular sampler was used to extract meat samples parallel to the muscle fibers, avoiding tendons. The length of each sample was at least 2 cm. The shear force was measured immediately after sampling.

### Myofibrillar fragmentation index

The MFI value was measured according to previously established methods [2]. Absorbance at 540 nm was measured using the UV spectrophotometer (UV1800; Salem Instruments). The MFI was calculated as 200 times the absorbance value.

### Scanning electron microscopy

The meat samples were fixed with glutaraldehyde solution (2.5%) and then dehydrated using ethanol solutions of varying concentrations. The dehydrated samples were subjected to critical point drying with CO_2_. After drying, the samples were mounted and coated with gold by ion sputtering. Upon completion of all procedures, the samples were observed under an environmental scanning electron microscope (GeminiSEM 300; Carl Zeiss).

### Myofibrillar proteins extraction

MPs were extracted according to previous method [12]. Meat samples were well homogenized (IKA, Dawson Springs) and washed twice in 20 mM phosphate buffer. Subsequently, they were washed with 0.1 M sodium chloride solution. Prior to the final centrifugation, the precipitate was filtered through a single layer of gauze (200 mesh) to remove connective tissue, and MPs precipitate was collected after centrifugation. The concentration of MPs was determined using the biuret method, as outlined by Gornall et al [13].

### Carbonyl content of myofibrillar proteins

The method of Li et al [14] was modified for carbonyl determination. Protein samples (500 μL of 5 mg/mL) were treated with 1 mL of 0.2% 2,4-Dinitrophenylhydrazine and incubated at 37°C for 30 minutes. After adding trichloroacetic acid, the mixture was centrifuged, and the pellet was washed with ethanol-ethyl acetate before dissolving in guanidine hydrochloride and incubating at 37°C for 20 min. The absorbance of the supernatant was measured at 370 nm (model P1; MAPADA Instrument), and the calculation formula was as follows:


(3) $$\text{Carbonyl content }(\text{nmol}/\text{mg protein})=\frac{A_{\text{sample}}-A_{\text{control}}}{22\times \text{L}\times {\text{C}}_{0}}\times 125\times 10^{5}$$

Where A: absorption; L: petri dish light diameter; C_0_: protein concentration.

### Sodium dodecyl sulfate-polyacrylamide gel electrophoresisof myofibrillar proteins

The concentrations of separation and concentrated gel were 10% and 5%, respectively. The protein was mixed with 5× Loading buffer (4:1), and denatured in a boiling water bath for 5 min. After cooling, 10 μL was loaded, with the stacking gel at 80 V and the separating gel at 120 V. The gel was stained for 30 min and decolorized until bands appeared clear. The images were acquired using the gel imaging system (model Azure 600; Azure Biosystems).

### Solubility and turbidity of actomyosin

AM was extracted following Martini et al [15]. The 2 mg/mL solution of AM was centrifuged for 10 min (4°C, 10,000×g). Protein solubility was calculated as the percentage of protein concentration after centrifugation relative to its initial concentration. The 1 mg/mL solution of AM was heated at 50°C for 30 min, and its turbidity was measured by absorbance at 600 nm.

### Ca**^2+^**-ATPase activity of actomyosin

The 2 mg/mL solution of AM was added to the Ca^2+^-ATPase reaction mixture (1:10 ratio) and incubated for 10 min at 25°C. The reaction was terminated with 1.0 mL of 10% trichloroacetic acid, and the supernatant was centrifuged. It was then mixed with ammonium molybdate and FeSO_4_ solutions, incubated for 2 min, and absorbance measured at 700 nm. Ca^2+^-ATPase activity was expressed as μmol (Pi)/min·mg pro at 25°C.

### Calpain-1 activity and caspase-3 activity

The calpain-1 and caspase-3 activity were measured using enzyme-linked immunosorbent assay kits (Shanghai Enzyme Link Biotechnology).

### Statistical analysis

Data analysis was performed using SPSS Statistics (SPSS) with two-way analysis of variance and Duncan’s multiple range test (p<0.05). Graphs were generated using Origin (OriginLab). All experiments were conducted in triplicate, and the results were reported as mean±standard deviation.

## RESULTS AND DISCUSSION

### pH

**Figure 1:**
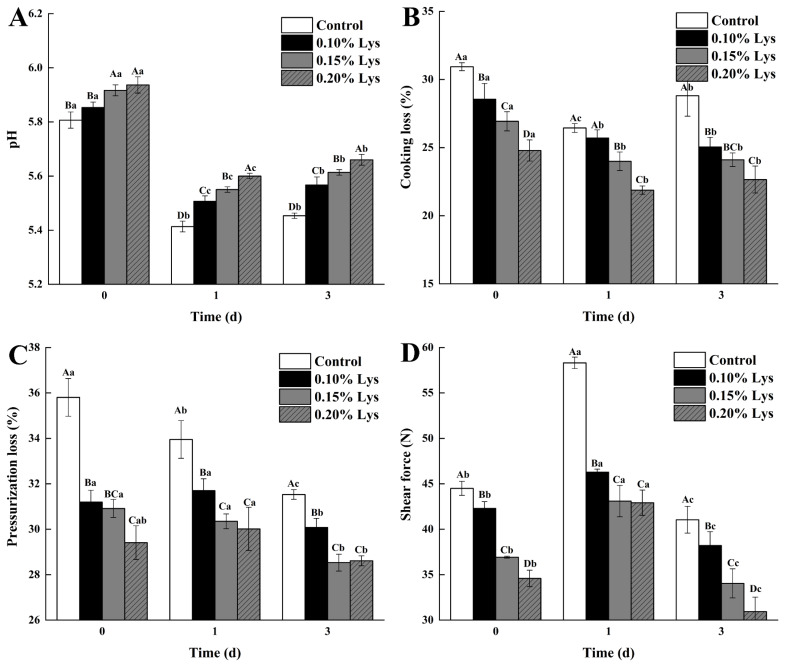
Effect of different treatments on pH (A), cooking loss (B), pressurization loss (C), and shear force (D). Control: meat samples were immersed in deionized water (pH = 7.0, 4°C) for 60 min; 0.10% Lys, 0.15% Lys and 0.20% Lys: meat samples were immersed in Lys solution (pH = 7.0, 4°C) at concentrations of 0.10%, 0.15% and 0.20%, respectively, for 60 min. The values are the means±SD. ^A–D^ Different capital letters indicate significant differences between treatment groups within the same aging storage time; ^a–c^ Different lowercase letters indicate significant differences between different aging storage time within the same treatment group (p<0.05) (n = 3). Lys, L-lysine; SD, standard deviation.

The pH changes in pork during postmortem aging affected the biochemical reactions and structural characteristics of muscle tissue, and the subsequent meat quality [2]. As shown in Figure 1A, the pH of samples initially decreased and then increased during the postmortem aging process, reaching minimum values of 5.41, 5.51, 5.55, and 5.60 at 1 day (p<0.05), respectively. As previously mentioned, pork on the first day of postmortem aging was in the rigor mortis phase [2]. During this phase, the cells underwent a hypoxic state, leading to the breakdown of glycogen into large amounts of lactic acid via anaerobic glycolysis, thereby lowering the muscle pH [16]. However, studies reported that a rapid decline in pH postmortem was detrimental to the development of desirable meat tenderness [17,18]. Notably, compared with control, the addition of Lys was found to increase the pH of pork in a dose-dependent manner during postmortem aging process (p<0.05). This might be due to Lys altering the secondary and tertiary structures of MPs, leading to the exposure of more acidic groups to interact with Lys, consequently changing the ionized functional groups of the protein and raising the pH of meat [10,11].

### Water-holding capacity

The CL and PL were key parameters reflecting WHC [2]. As shown in Figures 1B, 1C, both PL and CL generally showed a decreasing trend during postmortem aging. In comparison to Control, the presence of Lys decreased the PL and CL values, regardless of Lys concentration and aging time. Specifically, the 0.20% Lys treatment exhibited the lowest CL values, reaching at 21.87% and 22.66% at 1 day and 3 day (p<0.05), respectively. Similarly, the 0.20% Lys treatment showed the lowest PL value of 28.61% at 3 day (p<0.05), which was in line with the findings of Xu et al [2]. As mentioned above, the PL and CL values were decreased with the increasing Lys concentration (p<0.05), which could be attributed to the significant deviation of muscle pH from the isoelectric point of salt-soluble proteins, enhancing electrostatic repulsion between protein molecules and subsequently improving WHC [4,6,7]. However, a small increase in pH might have a limited effect on WHC. Previous studies found that Lys was effective in activating enzyme activities associated with the hydrolysis of MPs, increasing the interstitial space between fibres as well as between fibres and membranes to accommodate more water [2]. Zhang et al also observed that damaged myofibril structures as well as fuzzy Z-discs, M-lines, and I-bands were observed in Lys-treated chicken, which might be due to the fracture of myofibril fibres after MP degradation [19]. Fu et al further demonstrated that Lys had the ability to enhance WHC, which might be due to the amino group in the Lys molecule affecting the formation of hydrogen bonds in the protein gel [4].

### Shear force

Shear force serves as a reliable indicator of meat tenderness, with lower values indicating higher tenderness [11]. As displayed in Figure 1D, the shear force value of pork on the first day of postmortem aging was the highest (p<0.05), which was opposite to the change of pH values. This was likely due to the rapid decline in pH, which induced sarcoplasmic protein denaturation and filled the gaps between myofibrils, temporarily increasing mechanical resistance [20,21]. Meanwhile, the proteolysis of cytoskeletal proteins such as desmin and vinculin was not significantly activated until 24 hours postmortem [22], resulting in higher early structural integrity. Additionally, the rapid depletion of ATP led to the irreversible binding of myosin heads to actin (rigor mortis), further causing sarcomere shortening and increased shear force values [23]. Notably, the shear force values of Lys treatments were significantly lower than that of Control (p<0.05), suggesting that Lys had the potential to improve pork tenderness during the early postmortem period. Moreover, the shear force exhibited a dose-dependent reduction trend with the increasing Lys concentration. This was in agreement with previous studies, which found that Lys could improve the tenderness of marinated pork or chicken breast [11,24]. The decrease of shear force could be ascribed to two reasons: on one hand, Lys could interact with acidic amino acids of MPs through hydrogen bonding, leading to the unfolding of MPs and the concomitant dissociation of myofilament [25,26]. On the other hand, Lys could accelerate the degradation of cytoskeletal proteins (e.g. troponin-T, actin) [19,27], which might be resulting from the enhancement of endogenous protease activity (as proven below).

### Myofibrillar fragmentation index

MFI is commonly used to assess changes in the myofibrillar structure of muscle [27]. As shown in Table 1, a dose-dependent increase in MFI values was observed in the presence of Lys during postmortem aging process, (p<0.05), indicating that Lys might promote the breakdown of intermyofibrillar linkages and I-bands [28]. This finding was consistent with the study of Zhang et al [19], which showed an obscure I-bands and Z-lines of chicken breast with the addition of Lys. MFI also reflected the extent of postmortem proteolysis of the muscle cytoskeleton and associated proteins [29], which was primary induced by endogenous enzymes (e.g. calpain and caspase-3) in the early stages of aging [30,31]. Previous study indicated that Lys seemed to maintain calpain activity, which might in turn promote the degradation of MPs, ultimately leading to the increased MFI values and improved meat tenderness [19]. The effects of Lys on representative enzymes, such as calpain-1 and caspase-3, would be discussed in detail in the following sections.

### Scanning electron microscopy

**Figure 2:**
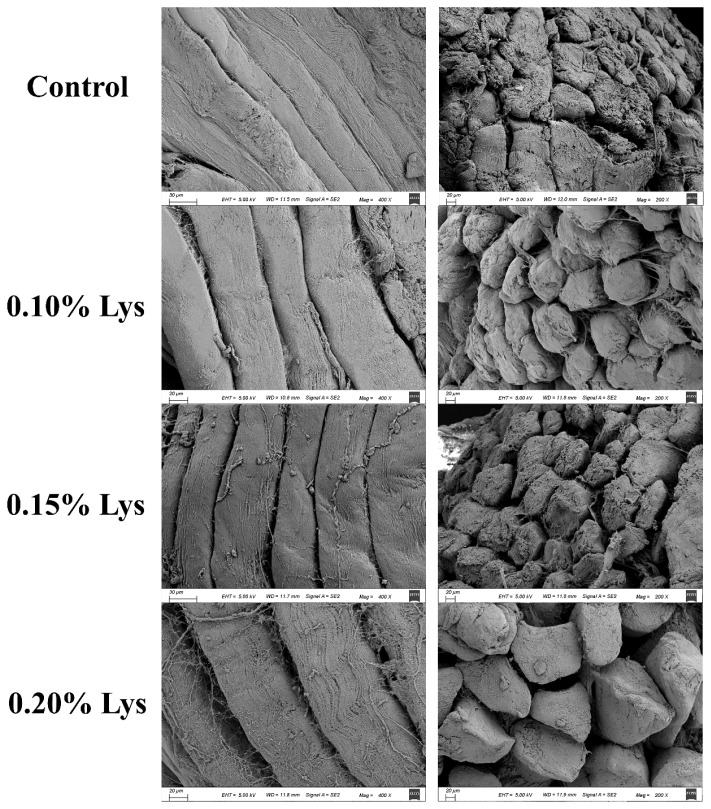
Microstructure of different treatments observed by SEM (3 d, left column: 400×, right column: 200×). Control: meat samples were immersed in deionized water (pH = 7.0, 4°C) for 60 min; 0.10% Lys, 0.15% Lys and 0.20% Lys: meat samples were immersed in L-lysine solution (pH = 7.0, 4°C) at concentrations of 0.10%, 0.15% and 0.20%, respectively, for 60 min. Lys, L-lysine; SEM, scanning electron microscopy.

Scanning electron microscopy could effectively characterize the differences in muscle fiber structure. Based on the analysis and discussion of meat tenderness and WHC during postmortem aging, we selected samples from the third day for micro-structural observation. As shown in Figure 2, the muscle fibers in Lys-treated samples exhibited significant structural changes compared to the control group. Specifically, as the concentration of Lys increased, the gaps between muscle fibers progressively widened, and the muscle fiber bundles transitioned from a dense to a loose state. This change might be attributed to the increase in pH (as shown in Figure 1A) that shifted away from the isoelectric point of proteins, which could create sufficient space between myofibrils to retain more water [7]. Additionally, we observed fiber breakage and degradation in the presence of Lys, especially under 0.20% Lys treatment. Previous studies found that Lys treatment disrupted the myofibrillar ultrastructure of meat, leading to the breakdown of the I-band and the disconnection between myofibrils [19,28]. These findings aligned with the results of shear force and MFI, suggesting that Lys could improve meat tenderness.

### Carbonyl content of myofibrillar proteins

Amino acid side chains in muscle proteins were highly susceptible to free radical attacks, leading to the formation of carbonyl groups. Thus, carbonyl content was a commonly used indicator to assess the extent of protein oxidation [32] and was also associated with protein functional properties such as WHC and texture [33]. During the postmortem aging process, the carbonyl content of pork significantly increased (Table 2) (p<0.05). This increase indicated that the amino acid residues on the protein side chains, containing free amino and imino groups, were oxidized due to free radical attacks during the aging storage period [25]. Notably, as the Lys addition increased, the carbonyl content in the muscle significantly decreased (p<0.05), suggesting that Lys effectively inhibited the conversion of polar amino acid residues into carbonyl derivatives, thereby reducing the degree of protein oxidation. This might be ascribed to the radical scavenging activity and iron chelating activity of Lys [10,25].

### Sodium dodecyl sulfate-polyacrylamide gel electrophoresis analysis of myofibrillar proteins

**Figure 3:**
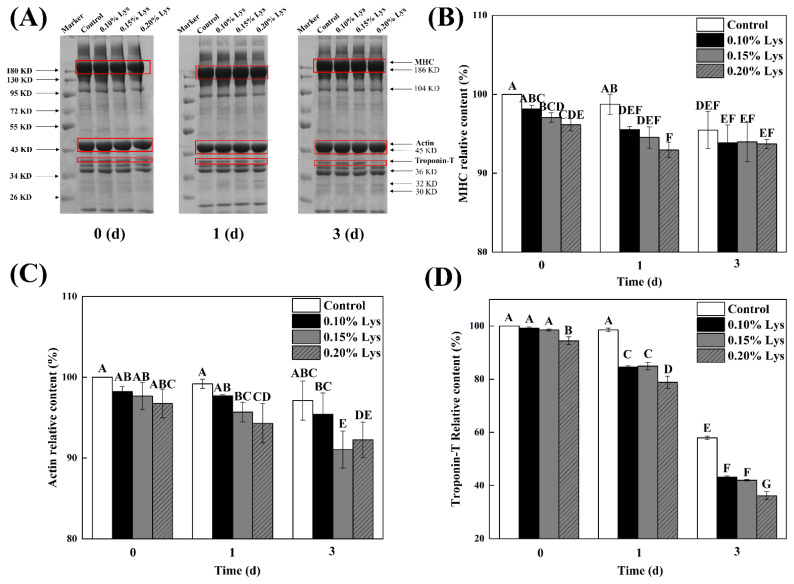
Effect of different treatments on sodium dodecyl sulfate-polyacrylamide gel electrophoresis (SDS-PAGE, A), myosin heavy chain (MHC, B), actin (C), and troponin-T (D) relative content. Control: meat samples were immersed in deionized water (pH = 7.0, 4°C) for 60 min; 0.10% Lys, 0.15% Lys and 0.20% Lys: meat samples were immersed in Lys solution (pH = 7.0, 4°C) at concentrations of 0.10%, 0.15% and 0.20%, respectively, for 60 min. The values are the means±SD. ^A–G^ Different capital letters indicate significant differences between different treatments (p<0.05) (n = 3). Lys, L-lysine; SD, standard deviation.

The electrophoresis results of different treatments were presented in Figure 3A, which displayed the characteristic pattern of MPs with three prominent bands corresponding to myosin heavy chain (MHC), actin, and troponin-T [17]. As shown in Figures 3B–3D, during the early postmortem period, the content of muscle proteins showed little change. As the postmortem aging process progressed, the relative contents of MHC, actin, and troponin-T in different treatments displayed a decreasing trend. As mentioned, the muscle pH gradually increased as the postmortem aging process continued, which helped the degradation of proteins (particularly actin and myosin) through enzymatic reaction. This, in turn, led to the loosening of the muscle fiber structure and increased tenderness of the meat [2,7]. It was worth noting that the band around 40 kDa gradually disappeared as time went by, which was attributed to the degradation of troponin-T [24]. Additionally, the relative contents of MHC and actin decreased as the Lys concentration increased (p<0.05). According to the previous studies, Lys facilitated the increase in myosin solubility by preferentially interacting with acidic and aromatic amino acid residues of the myosin molecules, thereby weakening myosin-myosin interactions [34]. Further, Lys could weaken the inter-molecular interactions of MPs, leading to the release of major MPs from muscle tissue and disruption of the structural integrity of the myofibrils [24,35]. Hence, Lys had the ability to promote the dissociation of the AM complex, which led to a more relaxed muscle structure and improved meat tenderness. Notably, the degradation of troponin-T significantly increased with increasing Lys concentration (p<0.05). As mentioned above, Lys increased pH by altering the secondary or tertiary structure of MPs and promoting the release of more acidic groups (Figure 1A), which might enhance calpain activity, thereby leading to the degradation of certain cytoskeletal proteins (such as troponin-T) [2]. Furthermore, Zhang et al [19] demonstrated that Lys could regulate the phosphorylation of structural functional proteins involved in meat quality development (such as α-actin-2) and energy metabolism proteins (such as Ca^2+^-ATPase and 6-phosphofructokinase), thereby promoting the instability of the skeletal muscle Z-disk and the glycolytic process, which contributed to the improvement of meat tenderness and textural characteristics.

### Solubility and turbidity analysis of actomyosin

The changes of solubility were used to assess the stability, folding state, and ability of proteins to remain dispersed in solution. As shown in Table 2, the solubility of Control first increased and then decreased with the increasing storage time (p<0.05), which was related to the pH changes in muscle. The pH of the muscle decreased during the early postmortem aging, bringing the pH value closer to the isoelectric point of proteins, thereby increasing intermolecular interactions and reducing solubility [36]. Moreover, compared with Control, the solubility of AM showed a dose-dependent increase in the presence of Lys (p<0.05). Lys could cause the unfolding of meat proteins, exposing the buried hydrophobic and SH groups, causing the depolymerization of myosin filaments, ultimately increasing protein solubility [26,37]. Higher protein solubility generally indicated better tenderness and WHC [2].

Turbidity was an important indicator reflecting the extent of protein aggregation and the quantity and size of suspended particles in solution [36]. Table 2 showed the changes in turbidity of AM under different treatments during postmortem aging. Turbidity of actomyosin decreased in a dose-dependent manner with increasing Lys concentration and was significantly lower than that of the control group (p<0.05). This was consistent with the result of solubility. The underlying reason might be that Lys bound to myosin molecules and disrupted intermolecular ionic bonds through electrostatic interactions, thereby inhibiting the aggregation of AM [38,39].

### Ca**^2+^**-ATPase activity of actomyosin

**Figure 4:**
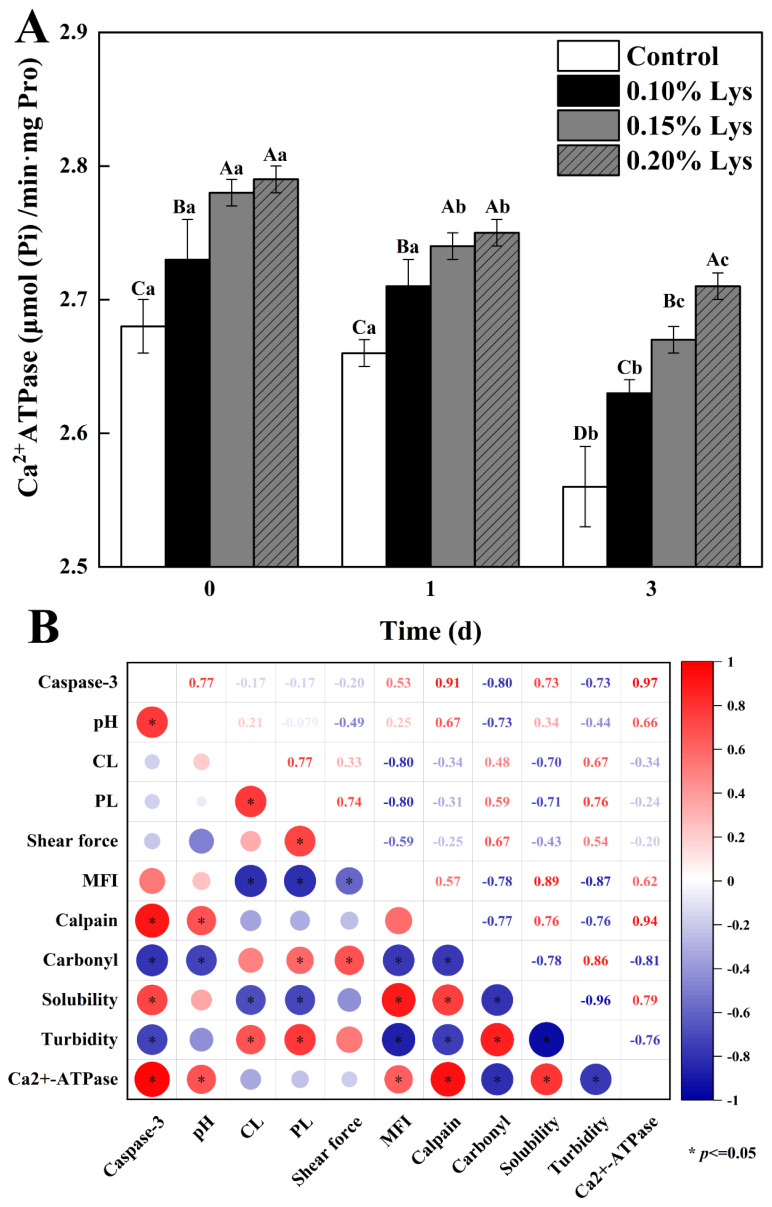
Effect of different treatments on Ca^2+^-ATPase (A) and correlation analysis (B). Control: meat samples were immersed in deionized water (pH = 7.0, 4°C) for 60 min; 0.10% Lys, 0.15% Lys and 0.20% Lys: meat samples were immersed in Lys solution (pH = 7.0, 4°C) at concentrations of 0.10%, 0.15% and 0.20%, respectively, for 60 min. The values are the means±SD. ^A–D^ Different capital letters indicate significant differences between treatment groups within the same aging storage time; ^a–c^ Different lowercase letters indicate significant differences between different aging storage time within the same treatment group (p<0.05) (n = 3). Lys, L-lysine; SD, standard deviation.

Typically, Ca^2+^-ATPase activity indicated the structural integrity of the active thiol (SH 1) in the myosin head catalytic center [36,40]. Changes of Ca^2+^-ATPase activity in AM was shown in Figure 4A. As the postmortem aging progressed, the Ca^2+^-ATPase activity gradually decreased (p<0.05), which was primarily due to the self-degradation and inactivation of enzyme during the early postmortem period [32,41]. The Ca^2+^-ATPase activity of samples at the 0 day was higher than that at other storage times, with the values of 2.68, 2.73, 2.78, and 2.79 μmol (Pi)/min·mg pro, respectively. Notably, the Ca^2+^-ATPase activity of the Lys treatments was significantly higher than Control regardless of aging time (p<0.05). In general, a higher Ca^2+^-ATPase activity indicated an accelerated rate of energy metabolism in the muscle [7]. The results indicated that Lys could accelerate the metabolism and shorten the postmortem ageing period. In addition, the higher structural integrity of SH 1 unit in myosin indicated that Lys might play a role in preventing actin binding to myosin head in the actin-binding cleft [7], thus facilitating the dissociation of AM and concomitant enhancement of meat quality. The effect of Lys on Ca^2+^-ATPase activity might be related to the interaction between the acidic residues of Lys and the aromatic residues of Ca^2+^-ATPase, which should be studied in the future.

### Calpain-1 activity and Caspase-3 activity

Calpain-1 was a calcium-dependent cysteine protease that affected meat tenderness by degrading key structural proteins in muscle during postmortem aging [42]. As shown in Table 1, calpain-1 activity was decreased during postmortem aging (p<0.05). This might result from the autolysis of calpain-1, which was triggered by the rapid post-slaughter pH drop [43,44]. In addition, calpain-1 activity exhibited an upward trend with the increasing Lys concentration. Similarly, Lys elevated muscle pH, which in turn increased calpain-1 activity [43]. Furthermore, the active center of calpain-1 consisted of cysteine, histidine and asparagine, and Lys had a positive effect on the polarization activation of the cysteine side chain [45]. During postmortem aging, calpain-1 played a crucial role in the hydrolysis of MPs, primarily located near the Z-line. It degraded actin and myosin, causing the dissociation between the I-band and Z-line in myofibrils, leading to myofibrillar fragmentation, which ultimately contributes to improved meat tenderness [42,46,47]. Therefore, Lys could increase calpain-1 activity, enhancing the dissociation and hydrolysis of key structural proteins, and ultimately contributing to improve meat quality.

Caspase-3 was a key protease involved in meat tenderization [36]. As shown in Table 1, caspase-3 activity exhibited a significant downward trend during postmortem aging (p< 0.05). This was consistent with the results for calpain-1, as the enzyme underwent self-degradation and inactivation in the early postmortem aging [41]. With increasing Lys concentration, caspase-3 activity showed an upward trend and was significantly higher than that in the control group (p<0.05). This suggested that Lys could activate caspase-3 activity. Du et al [48] found that caspase-3 was the initial trigger for accelerating muscle protein hydrolysis under catabolic conditions. Additionally, studies found that the rapid decline in muscle pH in the early postmortem aging significantly inhibited caspase-3 activity [49] and impacted the improvement of meat tenderness [17,18]. Moreover, Lys might interact with the cysteine residues in the active center of Caspase-3, thus activating Caspase-3 [45]. Therefore, Lys could initiate the caspase cascade, activating caspase-3 to hydrolyze MPs and subsequently improving meat tenderness [36].

### Correlation analysis

In this study, correlation analysis was used to investigate the relationships between protein characteristics, enzyme activity, and meat quality (Figure 4B). The results showed that pH was significantly positively correlated with calpain-1 activity, caspase-3 activity, Ca^2+^-ATPase activity, and solubility (p<0.05), while it was significantly negatively correlated with carbonyl content and turbidity (p<0.05). These findings were consistent with previous results [2], indicating a close relationship between pH, protein characteristics, and enzyme activity during postmortem aging. Additionally, significant correlations were found between protein characteristics and related enzyme activities, such as negative correlations between carbonyl content, turbidity, and calpain-1 activity, caspase-3 activity, and Ca^2+^-ATPase activity (p<0.05). The results of the correlation analysis suggested that Lys could influence the formation of final pork quality by regulating related enzyme activities and protein characteristics during postmortem aging.

## CONCLUSION

This study found that Lys improved pork quality by activating calpain-1 activity, caspase-3 activity, and Ca^2+^-ATPase activity during the postmortem aging, thereby promoting the degradation of MPs and the dissociation of the AM. Specifically, during postmortem aging, Lys altered the secondary and tertiary structures of MPs, exposing more acidic groups, which increased muscle pH and contributed to improvements in WHC and tenderness. Furthermore, the addition of Lys promoted MPs degradation and the dissociation of AM, as reflected in increased MFI values and the fragmentation and degradation of muscle fibers. Lys also unfolded meat proteins, exposing buried hydrophobic and sulfhydryl groups, leading to the depolymerization of myosin filaments and ultimately increasing protein solubility. Additionally, the Ca^2+^-ATPase activity in Lys treatments was significantly higher than in the control group, regardless of aging time (p<0.05). However, how Lys influences Ca^2+^-ATPase activity had not yet been specifically explored and requires further investigation. Lastly, with increasing Lys concentration, calpain-1 and caspase-3 activities in muscle were activated, enhancing the dissociation and hydrolysis of key structural proteins, thereby improving meat tenderness. However, this study had certain limitations, and future research should further explore the biochemical pathways involved in postmortem aging, such as glycolysis, and their related mechanisms influenced by Lys. Additionally, we also have acknowledged the importance of long-term observations (7–14 days) and the related studies will be conducted in the future.

## Figures and Tables

**Table 1:** The effect of different treatments on myofibrillar fragmentation index (MFI), calpain-1, and caspase-3 during different aging storage time

Time (d)	Sample ID	MFI	Calpain-1 (ng/mL)	Caspase-3 (μg/mL)
0	Control	71.47±1.81^Db^	14.59±0.43^Ca^	40.60±1.33^Ca^
	0.10% Lys	85.00±4.85^Ca^	16.04±0.53^Ba^	50.26±2.16^Ba^
	0.15% Lys	94.40±4.01^Bb^	16.20±0.12^Ba^	55.68±3.10^Aa^
	0.20% Lys	100.60±1.04^Ab^	17.46±0.42^Aa^	54.86±2.67^Aa^
1	Control	70.20±0.72^Cb^	14.52±0.16^Ca^	36.22±1.95^Cb^
	0.10% Lys	87.00±3.80^Ba^	15.29±0.28^Bb^	42.73±2.49^Bb^
	0.15% Lys	103.67±0.81^Aa^	15.22±0.14^Bb^	45.50±1.13^ABb^
	0.20% Lys	105.07±1.10^Aa^	16.36±0.35^Ab^	46.55±0.73^Ab^
3	Control	76.73±2.84^Ca^	13.07±0.21^Bb^	24.11±0.45^Dc^
	0.10% Lys	91.07±2.50^Ba^	13.20±0.03^Bc^	33.64±0.48^Cc^
	0.15% Lys	90.60±1.51^Bb^	14.96±0.44^Ab^	36.72±0.96^Bc^
	0.20% Lys	98.93±3.83^Ab^	15.56±0.69^Ac^	41.19±1.02^Ac^

The values are the means±SD. Control: meat samples were immersed in deionized water (pH = 7.0, 4°C) for 60 min; 0.10% Lys, 0.15% Lys and 0.20% Lys: meat samples were immersed in L-lysine solution (pH = 7.0, 4°C) at concentrations of 0.10%, 0.15% and 0.20%, respectively, for 60 min.

A–D Different capital letters indicate significant differences between treatment groups within the same aging storage time;

a–c Different lowercase letters indicate significant differences between different aging storage time within the same treatment group (p<0.05) (n = 3). Lys, L-lysine.

**Table 2:** The effect of different treatments on carbonyl, solubility, and turbidity during different aging storage time

Time (d)	Sample ID	Carbonyl (nmol/mg protein)	Solubility (%)	Turbidity
0	Control	13.59±0.23^Ab^	65.94±2.58^Db^	0.068±0.002^Aab^
	0.10% Lys	12.78±0.23^Bc^	81.64±1.00^Ca^	0.051±0.000^Bc^
	0.15% Lys	12.05±0.34^Cb^	88.67±1.02^Ba^	0.051±0.001^Bc^
	0.20% Lys	11.95±0.23^Cb^	90.35±0.46^Aa^	0.050±0.000^Bb^
1	Control	14.83±0.68^Aa^	70.96±0.72^Da^	0.066±0.001^Ab^
	0.10% Lys	13.76±0.17^Ba^	80.49±0.93^Ca^	0.054±0.001^Bb^
	0.15% Lys	12.98±0.10^Ca^	87.16±0.50^Ba^	0.053±0.000^Cb^
	0.20% Lys	12.47±0.10^Da^	89.24±0.37^Aa^	0.050±0.001^Dab^
3	Control	15.23±0.13^Aa^	68.89±0.54^Dab^	0.068±0.001^Aa^
	0.10% Lys	13.29±0.10^Bb^	77.66±1.32^Cb^	0.058±0.001^Ba^
	0.15% Lys	12.68±0.04^Ca^	81.40±0.98^Bb^	0.053±0.000^Ca^
	0.20% Lys	12.36±0.14^Da^	84.44±1.33^Ab^	0.051±0.000^Da^

The values are the means±SD. Control: meat samples were immersed in deionized water (pH = 7.0, 4°C) for 60 min; 0.10% Lys, 0.15% Lys and 0.20% Lys: meat samples were immersed in L-lysine solution (pH = 7.0, 4°C) at concentrations of 0.10%, 0.15% and 0.20%, respectively, for 60 min.

A–D Different capital letters indicate significant differences between treatment groups within the same aging storage time;

a–c Different lowercase letters indicate significant differences between different aging storage time within the same treatment group (p<0.05) (n = 3). Lys, L-lysine.
